# The evolutionary puzzle of cognition: challenges and insights from individual-based studies

**DOI:** 10.1098/rstb.2024.0123

**Published:** 2025-06-26

**Authors:** Daniel Sol, Alessandra Bateman-Neubert, Laura Noguer, Alex H. Taylor

**Affiliations:** ^1^ Institute of Evolutionary Biology (CSIC-UPF), 08003 Barcelona, Catalonia, Spain; ^2^ Centre for Ecological Research and Applied Forestries (CSIC-CREAF), Bellaterra, Catalonia 08193, Spain; ^3^ Institute of Neurosciences (INc-UAB), Universitat Autonoma de Barcelona, Bellaterra, Catalonia 08193, Spain; ^4^ Psychology, University of Auckland, Auckland 1010, New Zealand

**Keywords:** fitness, natural selection, phylogenetic-based analyses, problem-solving, spatial memory, Bogert effect

## Abstract

Cognition is widely believed to confer adaptive benefits, yet empirically demonstrating these benefits and understanding their evolutionary origin remains a significant challenge. Individual-based studies in the wild are essential for demonstrating that a cognitive trait is an adaptation. However, such approaches have so far yielded only partial evidence for the adaptive significance of cognition. Building on previous research, we highlight key challenges of individual-based studies that remain underappreciated and warrant further attention. These include the need for precise characterization of functionally relevant cognitive traits, a deeper understanding of heritable variation, more robust assessments of key fitness components across large cohorts and extended timescales, and clearer identification of the fitness benefits and selective pressures involved. We discuss how the lack of such comprehensive information limits our ability to fully evaluate how cognitive traits affect fitness, and to explore their demographic and evolutionary consequences. To bridge the gap between micro- and macroevolutionary processes, we also emphasize the need to better integrate individual-based research with broader population and species comparative analyses. By refining and expanding the approach, individual-based studies can deepen our insight into the evolutionary forces that have given rise to the remarkable diversity of minds across the animal kingdom.

This article is part of the Theo Murphy meeting issue ‘Selection shapes diverse animal minds’.

## 1. Introduction

**Figure 1.**
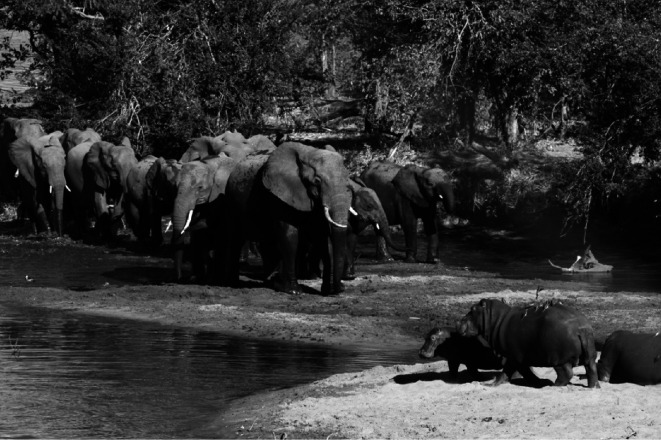
Cognition and decision-making in elephants. African elephants may consume up to 150 kg of food and drink 70−200 l of water daily. To meet these demands, they rely on mental maps of food patches and water sources across vast areas, and can recognize and consume around 90% of the plant species in their habitat [2,3]. In dry environments, some elephants have even learnt to dig for water using their trunks, and cover the holes with leaves to reduce evaporation. As elephants live in cohesive social groups, they can learn from experienced individuals to improve decision-making [4]. Given their long lifespans and the ecological impact of their foraging, regularly updating knowledge and refining skills is essential, as the conditions they currently face may differ significantly from those they will encounter in the future. Thus, flexible, experience-based decision-making is key for elephants to survive and reproduce in their harsh environments [3]. Photo: Daniel Sol.

Cognition—defined as the mental processes used by animals to gather, accumulate and utilize information about the external world [1]—underpins many decisions made by animals, such as where to live, what to eat or with whom to mate. As choices are fundamental to the way animals interact with their environment, and hence hold great potential to affect their survival and reproduction (see figure 1), we generally assume that cognition provides important adaptive benefits [5]. However, there is huge variation across animals in their ability to gather, accumulate and utilize information to make decisions [6,7]. For example, pigeons can master unexpectedly complex cognitive tasks and seem to use similar mental processes to crows [8]. Yet they acquire these skills more slowly, need more trials to make associations, and generalize less successfully to other problems than do corvids. Their brains are also less encephalized and contain fewer pallial associative neurons, features that may limit cognitive performance [8]. The existence of such variation might suggest that cognition is not equally adaptive in all contexts and/or may be shaped by costs, constraints or historical contingencies [8–10]. To substantiate that a cognitive feature is an evolutionary adaptation, it is fundamental that we understand how it affects fitness, and to identify the contexts in which these effects are beneficial and can evolve [11,12]. Understanding these issues is essential not only from an evolutionary perspective—to elucidate the evolution of cognition—but also to predict how animals will confront increasing threats such as climate change, overexploitation and habitat alteration [13,14].

The adaptive basis of cognition has traditionally been indirectly inferred from evidence suggesting that certain mental processes play critical roles in behaviours presumed to improve survival and/or reproduction, such as foraging decisions, mate choice, and communication efficiency [15,16]. One example is the use of mental maps by primates during foraging, which link cognitive traits to the benefits of exploiting spatially and temporally patchy food resources [17]. Further evidence for the adaptive basis of cognition comes from comparative studies showing that variation in cognitive performance across species predicts their demography in maladaptive scenarios, particularly in contexts where behavioural plasticity plays a crucial role. For instance, avian species with a higher propensity for innovation in foraging have been found to be more successful at thriving in new environments and avoiding extinction caused by habitat alterations [13,18,19]. Recently, there has been a growing interest in using individual-based approaches (*sensu* [20]) to directly address how cognition affects fitness and evolves in wild populations (reviewed in [21]). A compelling case comes from research on spatial cognition in mountain chickadees (*Poecile gambeli*). During the harsh winter months, these birds depend heavily on hiding and retrieving food caches, which suggests that spatial cognition is critical for their survival. Research by Pravosudov and colleagues [22] provides evidence in support of this hypothesis, showing that individuals who perform better in spatial memory tests are more likely to survive the winter. The genetic basis for variation in spatial memory—a critical aspect in demonstrating its adaptive nature—and its neural basis have also been identified [22]. The research on mountain chickadees, along with other similar field studies (reviewed in [21]), highlights that investigating the adaptive significance of cognitive performance in wild individuals is both feasible and highly insightful.

While individual-based studies pave the way for a more mechanistic understanding of cognition as an adaptation, the approach is fraught with challenges. Previous reviews have highlighted some of these challenges—particularly the difficulties of accurately characterizing and quantifying cognitive variation among individuals [11,12,23,24]. However, other crucial aspects—such as the trait’s functional relevance, potential trade-offs with other traits, effects on overall fitness, demographic implications and evolutionary potential—have received comparatively less attention. In this review, we argue that overlooking these aspects has significant implications for individual-based research on cognition, ultimately narrowing the range of questions we can address to demonstrate that a cognitive trait is adaptive (see table 1). Additionally, we emphasize that integrating individual-based data across populations and species is crucial for linking contemporary evolutionary processes with historical ones [25], thereby bridging micro- and macroevolutionary patterns and enhancing our understanding of the diversification of the animal mind. While our review builds on earlier arguments about how to test the fitness benefits of cognition [11,12,23,24], it seeks to enrich the debate by placing these arguments within a broader evolutionary ecology perspective.

**Table 1.** Central questions to understand the adaptive nature of a cognitive trait.

1.	**What is the function of the cognitive trait?** What role does the trait play in acquiring information, making decisions and improve behavioural performance? Does the trait provide an advantage in specific ecological or social contexts? Are there trade-offs associated with other traits?
2.	**What are the fitness consequences of the trait?** How does the trait affect individual survival or reproduction? Are there costs associated with the trait?
3.	**Is there heritable variation in the trait?** What proportion of the trait's variation is due to genetic factors versus environmental influences? How does the trait integrate into an organism's overall phenotype?
4.	**What are the fitness consequences of variation in the trait?** Does variation in the trait generate differences in fitness between individuals from the population? How does this, in turn, affect the population’s growth and long-term persistence?
5.	**How does the trait evolve?** Does the trait respond to selection? Do evolutionary constraints, such as genetic correlations or pleiotropy, affect evolutionary responses? How do non-adaptive processes, such as genetic drift or gene flow, influence trait evolution?
6.	**What selective pressures shape the trait?** What social or ecological factors can drive the evolution of the trait? How do these pressures vary across space and time? Is the trait currently optimized for the environment in which it occurs?
7.	**What is the evolutionary history of the trait?** Are the selective pressures currently operating similarly to those operating in the past? How has the life history of organisms constrained the evolution of the trait? Is the trait an exaptation?

## 2. Why are individual-based studies essential to demonstrate that a trait is an adaptation?

**Figure 2.**
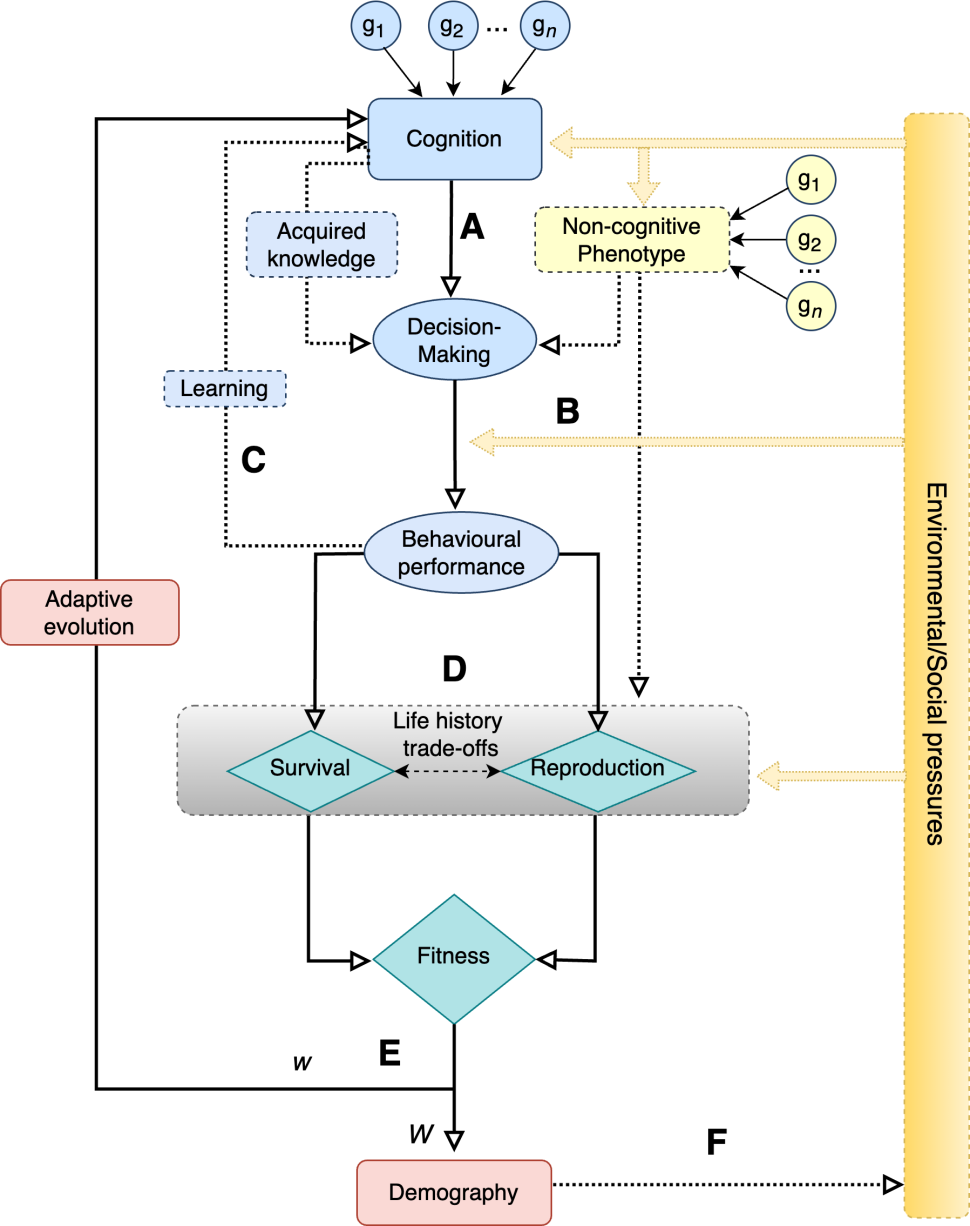
Framework for understanding the fitness consequences of cognition. Animal decision-making is primarily guided by cognitive processes in the brain, which are shaped by a variety of genes (g_1_, g_2_, …, g_*n*_) and environmental factors (A). Decisions are further influenced by the acquired knowledge retained in the memory, and by non-cognitive traits such as motor abilities and emotional responses. When faced with environmental or social challenges (e.g. a food shortage), the decisions made by the animal can generate behavioural responses that mitigate these challenges (e.g. adopting a new foraging technique) (B). The new knowledge and skills can be stored in memory, enhancing future decisions and behavioural responses (C). Behavioural performance directly influences one or more fitness components (e.g. reducing adult mortality caused by food shortages) (D). The overall impact on fitness also depends on the effect of other phenotypic traits and life-history trade-offs. For instance, improving survival can come at the cost of reducing fecundity. Cognition is considered adaptive if it enhances overall fitness (E). Specifically, the trait must, on average, enable individuals to leave at least one offspring to replace themselves—this is the concept of absolute fitness (*W*), commonly used in population ecology and defined as the total number of offspring an individual contributes to the next generation. For cognition to evolve, it must also confer a fitness advantage to the individual relative to other individuals from the population, described as relative fitness (*w*), a concept central to evolutionary biology. Demographic and evolutionary consequences, in turn, influence the alignment of the population to its environment, either by improving the average performance of individuals or by modifying the environment. For example, improved fitness might lead to higher population density, intensifying competition for resources such as food (F). In the diagram, solid lines represent the primary pathways linking cognition to demographic and evolutionary outcomes, while dotted lines highlight factors that influence these pathways. Double arrows indicate trade-offs.

A trait is considered an adaptation for a specific function if it has evolved through natural selection to perform that function and, as a result, enhances the organism’s fitness [26,27]. Individual-based studies—which track the development and life histories of identifiable animals [20]—are essential for demonstrating this (figure 2). First, these studies enable researchers to assess the extent to which a trait influences how individuals survive and reproduce across different life stages. By linking these key life-history events, they help estimate the demographic consequences of trait variation and evaluate how well a population is adapted to its current environment. Second, individual-based data collected over multiple generations allow researchers to estimate the genetic variances and covariances of traits, as well as their degree of plasticity, using a quantitative genetics approach [20,28]. By combining heritability and fitness measures, this approach also allows us to determine whether a trait is under direct selection—rather than being influenced by genetic correlations with other traits—and to what extent selection is likely to drive adaptive evolution [29]. Thus, individual-based studies provide a comprehensive framework for understanding the relationships between phenotype, performance, fitness, demography and evolution (figure 2). In the following sections, we discuss the critical challenges of the individual-based approach—challenges related to measuring variation in cognitive performance among individuals, disentangling heritable from non-heritable effects, estimating fitness consequences, and predicting both microevolutionary and macroevolutionary outcomes—while outlining current solutions and proposing future research directions.

## 3. Measuring variation in cognitive performance

Understanding the evolution of cognition through an individual-based approach largely relies on accurately assessing cognitive differences between individuals [12]. This is not an easy task. Cognition cannot be observed directly, but is inferred from behavioural performance [11,12,30]. Even if the behaviour has a genetic basis, its expression is still environmentally induced and subject to emotional, motivational and stochastic influences [31]. Thus, a major challenge lies in designing experimental assays that isolate cognitive traits from noise and confounding factors [12,15,32].

Currently, there exist a number of psychometric tests that allow us to accurately estimate performance of individuals in a variety of cognitive functions—such as inhibitory control, associative learning or spatial memory [12]. Psychometric tests work well in the controlled conditions of the laboratory, even allowing for the evaluation of the consistency of the cognitive measures within individuals [16,33]. For example, Madden *et al.* [16] used this approach to assess the performance of pheasant (*Phasianus colchicus*) chicks in acquisition and reversal learning of spatial and colour cues, before releasing them to follow their fates in the wild. Conducting similar experiments in the field is considerably more complex, primarily owing to difficulties in controlling for confounding factors such as motivation, emotional responses and social interactions. However, recent studies have demonstrated that psychometric tests can also be effectively implemented in the field. For example, Ashton and co-workers [34] investigated the fitness benefits of cognition in Australian magpies (*Gymnorhina tibicen*) through field-based cognitive tests designed to measure inhibitory control, associative learning, reversal learning and spatial memory, while statistically controlling for individual differences in motivation by including body condition as covariate in the models. The potential to conduct psychometric tests in natural settings is an exciting advance as field experiments better reflect cognitive functions relevant to the animal under realistic and less stressful conditions [15].

Although focusing on specific cognitive traits is ideal for understanding cognitive evolution—especially by contrast to broader problem-solving abilities that involve both cognitive and non-cognitive components [12,35]—this targeted approach can sometimes obscure the ecological relevance of those traits. Few would dispute that a spatial memory test is relevant for food-hoarding animals, which need to remember the locations of their caches [36,37]. However, cognition encompasses far more than solving problems in specific contexts. A primary function of cognition is to support flexible decision-making across a wide range of tasks and situations, including novel ones. Measuring this type of domain-general cognition is challenging, as it reflects an emergent property arising from the interaction of multiple underlying cognitive traits [31]. This difficulty is currently addressed by employing a battery of psychometric tests and combining the outcomes into composite measures of ‘general cognitive performance’ [23,32,34]. Although the use of a battery of psychometric tests has significantly advanced our understanding of cognitive evolution, interpreting ‘general cognitive performance’ remains challenging. This difficulty arises from the fact that performance depends on the specific traits included in the assessment—traits that can be measured with varying degrees of accuracy and are influenced to different extents by factors such as genetics, motivation, emotional state, social dynamics and even chance [12,38]. The proximate causes of the integration between the traits—such as shared genetic, hormonal or developmental pathways—and the consequences of the resulting emergent properties for decision-making processes also require further attention.

Psychometric testing—particularly when involving a battery of tests—is time-consuming, logistically complex and financially demanding. As a result, most studies investigating the adaptive function of cognition rely on small sample sizes [21], which conflicts with the large samples required for robust quantitative genetics and selection analyses (see §§4–6). Nevertheless, research by Cole *et al.* [33], Madden *et al.* [16] and Fitchtel *et al.* [39], among others, demonstrates that large-scale testing is indeed feasible. In addition, technological advancements—such as improved tracking devices, high-resolution cameras and portable electronic tools—are facilitating the standardized testing of large numbers of individuals, while also enabling the automated collection of data [40]. For example, radio-frequency identification (RFID)-based feeders can be used to conduct various visual cognitive tests, such as associative learning and spatial memory assays [22,41], with minimal human intervention. These methods offer unprecedented opportunities to estimate cognitive performance in the wild across numerous individuals, over extended periods and across large geographical areas.

## 4. Assessing heritable variation in cognition

For a trait to qualify as an adaptation, the trait must be heritable, meaning that genetic instructions can be passed on to the next generation. The genetic basis of some cognitive traits has been well established based on laboratory studies [38,42,43]. As cognitive performance is generally assumed to depend on many genes, this has usually been investigated using quantitative genetic approaches. For instance, Langley *et al.* [38] used an ‘animal model’ approach to show that, in pheasants, discrimination learning and inhibitory control exhibit moderate heritability and reduced genetic correlation with each other. While laboratory studies offer controlled conditions that help isolate genetic contributions, they often fail to replicate the complexities of natural environments. The few attempts to date to estimate the amount of heritable variation of cognitive traits in wild populations have yielded mixed results [36,44]. For example, while spatial memory performance in food-hoarding mountain chickadees has been shown to be highly heritable [36,44], learning does not appear to exhibit significant heritable variation either in Australian magpies [45] or in toutouwais (*Petroica longipes*) [46].

Accurately estimating how much of a trait’s variation is heritable is not easy in general, requiring genotyping many individuals of different generations to build robust pedigrees [38]. In the field, additional challenges arise owing to the difficulties of controlling for environmental effects, which can either inflate or reduce heritability estimates. As heritability reflects the proportion of phenotypic variance attributable to additive genetic variance, increased phenotypic variance will lead to an underestimation of heritability. This means that detecting significant heritability can be challenging when measures of cognitive performance are noisy, for example because some individuals are more motivated or fearful than others in engaging with the experimental apparatus [46]. Conversely, heritability can be overestimated when the resemblance of individuals with their parents does not result from shared genes, but rather from growing up in similar environments or acquiring knowledge through cultural transmission [47]. As cognitive performance can be shaped by development, past experiences and acquired knowledge, there is a serious risk of confounding heritable variation with permanent effects that are actually environmentally induced. Such biases can be reduced by accounting for maternal effects, permanent environmental influences and indirect genetic effects within the ‘animal model’ framework [48]. Additionally, cross-fostering experiments—where offspring are exchanged between different parents—help disentangle genetic and environmental contributions to trait development [47]. Owing to the challenges of obtaining adequate sample sizes and controlling for environmental factors in wild populations, our current understanding of the genetic variation in cognitive traits within these populations remains limited.

## 5. Estimating the fitness benefits of cognition

When consistent differences in cognitive performance exist among individuals, we can explore how these differences impact their fitness. At the individual level, fitness is primarily—though not exclusively—defined by the number of offspring an individual produces over its lifetime (*lifetime reproductive success*), which largely determines how many genes are passed to the next generation. Assessing this requires long-term monitoring of reproductive success across many individuals [28,29]. In practice, most studies examining fitness consequences of cognition are relatively short in duration (typically 2−3 years). With some exceptions, they also typically involve reduced sample sizes. Detecting the fitness effects of cognition may be difficult when measured over short periods—particularly if selection pressures are weak, fluctuating or result from rare, high-impact events—or when based on reduced sample sizes, as measures of cognition tend to be noisy [23].

The chances of detecting fitness effects of cognition also depend on the ecology of the species. In social species, many individuals can benefit from the decisions made by a few informed or skilled individuals [38], for example through public information or social learning. This should reduce fitness variation, and hence the probability of finding fitness effects of cognition. Likewise, the potential of the species to construct their own niche may affect variance in fitness among individuals. This is obvious in human evolution, where advances in hygiene and medicines have reduced the mortality of infants, altering the correlation with fitness of traits such as body size and age of first reproduction [49].

It is also important to note that, under evolutionary equilibrium, the fitness components with the greatest impact on overall fitness tend to exhibit reduced variation [50], complicating the detection of fitness effects. For instance, if cognitive flexibility (e.g. innovativeness) evolved as a defence against extrinsic mortality factors (e.g. food shortages), as proposed by the cognitive buffer hypothesis [5,51], the resulting reduction in survival variability could obscure any detectable relationship between survival fitness and the underlying cognitive traits. An implication is that such fitness consequences are more likely to be detectable in situations of evolutionary disequilibrium, such as during colonization events or when an animal is exposed to environmental changes. Since cognition involves gathering information to improve decision-making, the re-introduction of a species to a new environment can also offer valuable insights into the fitness consequences of cognitive traits [16,52].

Despite the difficulties, there is growing evidence that cognition can enhance some fitness components (reviewed in [21]). For instance, in Australian magpies [34], domain-general cognitive performance was positively correlated with reproductive success. In mountain chickadees and wild grey mouse lemurs (*Microcebus murinus*), the fitness component that predicts cognition is survival [39]. Although cognition is expected to entail costs, studies reporting negative or null associations with fitness remain relatively rare. One exception is in common pheasants (*Phasianus colchicus*), where chicks that were slower to reverse previously learned associations had higher survival rates [16]. The scarcity of negative or null findings could, in part, reflect a publication bias [21].

Even when some studies report evidence for a positive effect of cognition on fitness, the underlying reasons are generally uncertain. Of course, we can expect that spatial memory is relevant in food-hoarding birds that have to remember where they hide all their caches to avoid starving when snow reduces food availability [36]. In other cases, however, how the target cognitive trait influences the way individuals interact with the environment remains more uncertain. The focus on cognitive traits, rather than ecologically relevant behaviours, may add to this uncertainty. For example, the negative relationship between reversal learning speed and individual survival observed in pheasants is puzzling, given that reversal learning is typically considered a proxy for behavioural plasticity [16]—an ability presumed to aid in coping with unfamiliar environments. However, what if reversal learning is not a reliable indicator of behavioural plasticity? What if the ability to rapidly form new associations—crucial for coping with unfamiliar environments—is more important for survival than inhibiting previously learned ones, yet the formation of such strong initial associations makes it harder to later reverse them? The general lack of functional context for interpreting the ecological relevance of cognitive traits presents a critical gap in our understanding of their fitness consequences, stressing the need to pay more attention to how cognition enables individuals to interact with their environment.

## 6. Understanding how cognition can evolve

The covariation between a phenotypic trait and fitness determines the strength of selection. By combining measures of selection strength (e.g. selection differentials or gradients) with estimates of heritable variation, we can use tools such as the Breeder’s Equation or the Robertson–Price Identity to analyse how selection drives adaptive evolution [53]. For example, the Breeder’s Equation posits that the evolutionary response of a trait is proportional to the strength of selection and the amount of heritable variation, allowing prediction of whether a trait will evolve over time and how much it will change. These analytical tools can enable researchers to assess the relative contributions of selection versus other evolutionary forces (e.g. genetic drift and gene flow), explore the factors that maintain variation in cognition despite directional selection, and investigate why an evolutionary response may be constrained even in the presence of selection.

The potential of the above tools remains largely to be exploited to study evolutionary responses of cognition to selection. One reason is that, while a growing number of studies show that individual differences in cognitive performance can result in differential fitness, in most cases the heritability of the cognitive traits remains to be demonstrated [39]. The short-term approaches to estimate fitness (§5) also limit the utility of these tools. In their studies of New Zealand robins (*Petroica longipes*), for instance, Shaw *et al.* [54] reported that males with better spatial learning and memory produced more fledglings and independent offspring per nesting attempt, but not over the entire season—suggesting no evidence for strong active selection. The narrow focus on particular fitness components is another aspect that can limit progress, as a positive effect on a fitness component (e.g. reproductive success) may come at the expense of another component (e.g. survival, through the so-called cost of reproduction [55]). In great tits (*Parus major*), for example, Cole *et al.* [33] found that problem-solver females produced larger clutches than non-solvers, yet they were more likely to desert their nests, leading to little or no overall selection on problem-solving performance. Focusing on a specific fitness component is informative only if the component is directly affected by the cognitive trait and is a major determinant of lifetime reproductive success. Among female baboons, for example, reproductive lifespan accounts for 50% of the variation in lifetime fitness [56,57]. This means that, for a female, dying young has a greater impact on fitness than delaying or skipping reproduction, implying that survival may be a more accurate proxy for fitness than fecundity (but see §8). Thus, the fitness measure used in a study should be determined by a life history logic, not merely by ease of access.

We can also envision scenarios where it may be difficult to detect evolutionary responses, even when the trait shows heritable variation and provides fitness benefits. First, if the trait is close to the optimum, and gene flow is reduced, we should expect stabilizing selection rather than directional selection. When selection acts against extreme phenotypes, detecting selection requires larger sample sizes to include enough individuals at the tails of the distribution [58]. Second, if selection pressures are fluctuating or arise from rare, high-impact events, then detecting evolutionary responses may require monitoring many generations [59]. Third, natural selection acts on individuals as a whole, not on isolated traits. Cognitive traits can be genetically or phenotypically correlated with each other or with traits such as social status or body size, which may themselves be under selection. Under directional selection, a trait may respond positively, negatively or not at all, depending on the strength and direction of its genetic and phenotypic correlations with other traits—even if it is not directly targeted [60,61]. Finally, individuals have the potential to reduce the strength of selection by selecting habitats or resources that better match the phenotype of the individual [62], a phenomenon known as the Bogert effect [62–65]. Given the scarcity of studies, it remains unclear whether any of these scenarios significantly limit our ability to detect evolutionary responses in cognitive traits.

To further complicate things, passing genes on to future generations is not just about producing offspring; it is also about facilitating them to produce grand-offspring that, in turn, produce great-grand-offspring (and so on). In cetaceans and primates, for example, postmenopausal females may play a vital role by passing on their knowledge to help younger generations survive and reproduce [66]. Helping close-relatives, with which the individual shares many genes, to survive and recruit in the population may influence evolutionary dynamics by creating indirect links between cognition and fitness.

Given the numerous challenges involved, it is unsurprising that research on evolutionary responses to selection in cognitive traits remains scarce. One notable exception is the work by Pravosudov and colleagues [22] on food-hoarding chickadees. In these birds, spatial memory and hippocampal size differ across populations exposed to varying degrees of winter severity. Common garden experiments—rearing nestlings from different populations under identical conditions—have shown that these differences are likely genetic. Along with evidence that spatial memory shows heritable variation and influences survival and longevity [36,44], these findings support the view that natural selection adapts spatial memory to the demands of cached food [22]. Additional studies that integrate analyses both within and across populations are needed to broaden generality and deepen our understanding of the selective forces shaping the evolution of cognitive traits under diverse selective regimes.

## 7. Exploring population implications of the covariation between cognition and fitness

**Figure 3.**
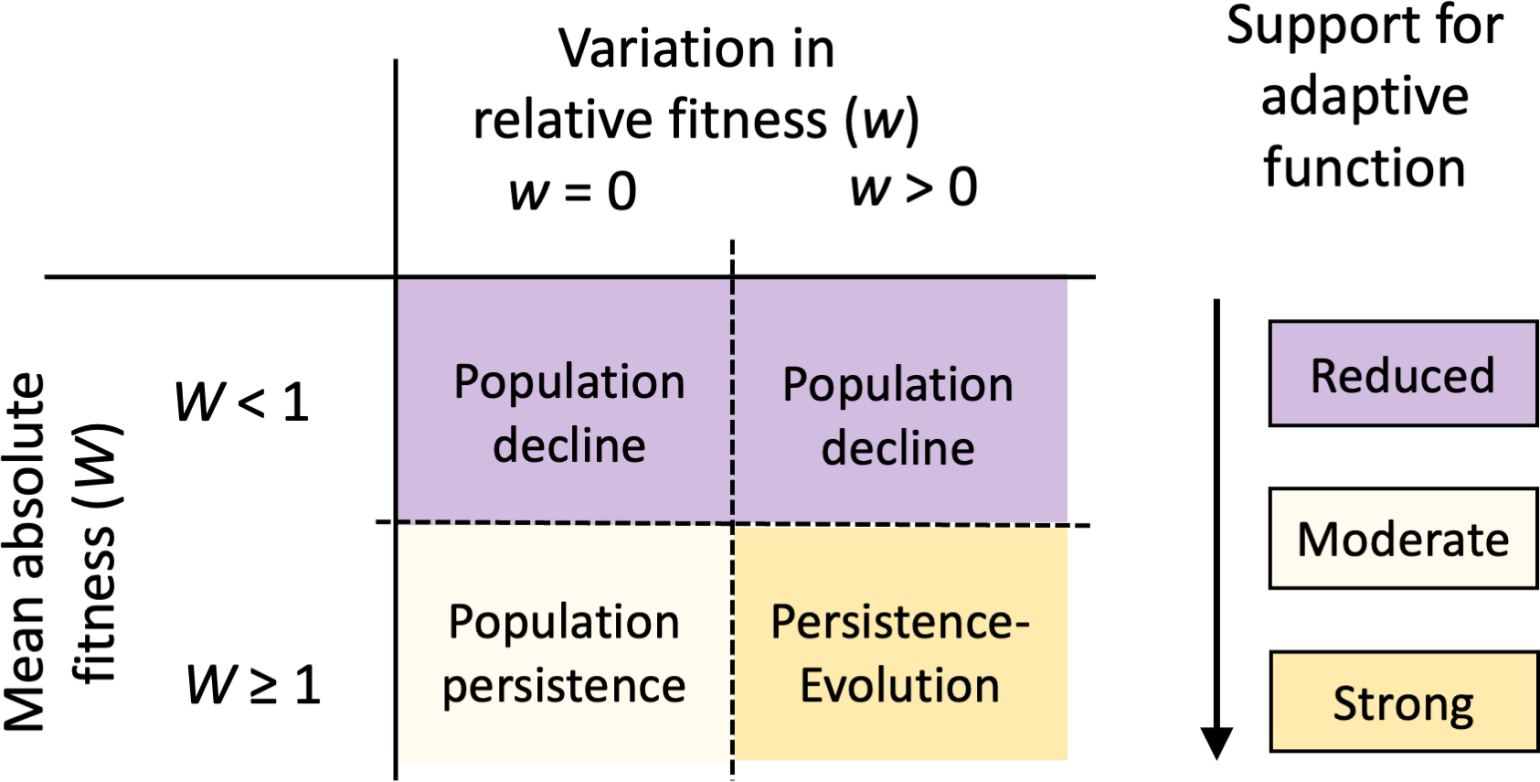
Importance of relative and absolute fitness to understand the adaptive nature of a trait. Absolute and relative fitness are interconnected, as natural selection acts on genetic variation among individuals to optimize the alignment between an organism’s phenotype and its environment. By enhancing this match, selection increases the average absolute fitness of a population. However, understanding whether a trait is adaptive requires distinguishing the implications of absolute and relative fitness. From the perspective of absolute fitness, a population that is stable or growing (*W* ≥ 1) can be considered well adapted to current conditions, while a declining population (*W* < 1) suggests maladaptation. Therefore, both absolute and relative fitness can provide evidence of a trait’s adaptiveness, but only in scenarios where the population is stable or growing. A declining population cannot be considered well adapted to present conditions, even when the individuals with the trait exhibit higher relative fitness (variation in *w* > 0). This does not imply that the trait is inherently non-adaptive: a trait may have evolved as an adaptation even if its original function is no longer critical. However, in such cases, there is less direct evidence to support its adaptive nature.

Our discussion thus far has focused on fitness differences among individuals—reflecting the concept of relative fitness used in evolutionary theory (figures 2 and 3). However, cognition can be essential for fitness even in the absence of variation among individuals (see §8). For instance, when a species faces challenges that are well within its cognitive capacity, all individuals may be equally capable of solving them, resulting in little to no cognitive variation. Conversely, even when some individuals outperform others cognitively, this may not be sufficient to maintain population persistence if cognitive performance remains inadequate—an indication of maladaptation. Therefore, relying solely on relative fitness may not provide a complete picture of the adaptive value of cognitive traits. It is also important to consider absolute fitness—the number of offspring an individual contributes to the next generation [67]—a key concept in population biology (figures 2 and 3).

While it may seem obvious that a growing or stable population is better adapted to current conditions than one that is declining [67], the need to consider estimates of absolute fitness (in addition of relative fitness) remains underappreciated in studies of cognition. Interestingly, there are a number of factors that may cause conflicts between relative and absolute fitness, including sexual selection, density-dependent effects and human-induced environmental changes [68]. One particularly intriguing factor is sexual selection, which has been suggested as an important driver of cognitive evolution [69]. In general, a preference for mates with exceptionally elaborated traits can increase reproductive success while potentially reducing viability, leading to higher relative fitness but lower absolute fitness [67]. However, if females select mates based on superior cognitive abilities [69], this could result in increases in both relative and absolute fitness. Another factor that can generate conflicts between relative and absolute fitness is a sudden alteration of the environment [69], such as those caused by human activities. These alterations can move the phenotype of many individuals far from the optimum, potentially increasing variation in fitness among individuals while reducing their average absolute fitness. Examining demographic effects through individual-based approaches, and investigating conflicts between absolute and relative fitness, may further illuminate the adaptive significance of cognition.

## 8. Linking microevolutionary and macroevolutionary processes

The phenotype of any species has in part been inherited from the ancestors and in part evolved subsequently to align with its current environment. Our discussion thus far has centred on how cognition enhances fitness and evolves under present-day conditions. While an individual-based approach may connect the process of selection to longer-term evolutionary trends, their predictions are limited to microevolutionary scales. Most striking neural and cognitive divergences evolved millions of years ago [5,70], and hence cannot be fully understood through a microevolutionary approach.

Since evolution alters the relationship between organisms and their environments, we cannot assume that the selective pressures acting in the past are the same as those operating today. For example, if past selection reduced variation in the most critical fitness component, contemporary selection may currently act more strongly on other components that remain variable [49]. Take, for instance, the low fecundity, prolonged development, delayed sexual maturation and high longevity typical of animals with advanced innovative capacities, such as primates and corvids [10]. For species with such slow life histories, longevity is expected to be a major determinant of lifetime reproductive success. However, in contexts where adult survival is already very high, further increases in survival may offer limited gains in relative fitness. Instead, ensuring the survival of their relatively few offspring may now be more consequential, potentially shifting selection towards traits like extended and enhanced parental care. Crucially, this shift may occur even if enhanced parental care was not the original driver behind the evolution of enhanced innovativeness. A historical perspective is therefore essential for understanding how cognitive traits evolve and diversify. Comparing species through a phylogenetic comparative framework provides such a perspective.

The phylogenetic comparative approach has a long-standing tradition in cognitive ecology, and has offered important inspiration and insights for individual-based analyses. For instance, evidence that innovativeness and encephalization are more developed in species frequently facing seasonal changes in their environment [71] suggests that selection for cognitive plasticity should be more important in such type of environments. This prediction has been validated in individual-based studies of mountain chickadees [44]. The phylogenetic comparative approach also offers powerful modelling methods for interpreting current differences in cognition among species as outcomes of past evolutionary processes, allowing, for instance, estimation of ancestral states, phylogenetic constraints, reconstruction of changes in selective regimes, testing the adaptive basis of a trait, and investigation of coevolving traits [27,72,73]. Such retrospective approaches have been used, for example, to investigate why migratory species tend to show a lower propensity for innovation and possess less encephalized brains compared with resident species that reproduce in the same seasonal environments [74]. By combining phylogenetic reconstructions with Ornstein–Uhlenbeck evolutionary models [27], Sayol *et al.* [73] found evidence consistent with selection favouring increased encephalization in species that remain and reproduce in highly seasonal environments, and selection favouring reduced encephalization in those that migrate to avoid harsh winter conditions. The use of similar phylogenetic approaches to study the adaptive significance of cognition traits has not yet been fully integrated in individual-based studies, although it holds considerable promise. Such methods could help address key questions, such as whether a cognitive trait evolved specifically for its current function—making it a true adaptation—or whether it originally served a different purpose and was later co-opted for its present role, in which case it would be considered an exaptation. These approaches can also illuminate the extent to which cognitive traits have been shaped by ancestral constraints [27], offering valuable insights into why certain abilities—such as self-recognition or the attribution of mental states to others—appear to be restricted to only a few species.

We highlight two major challenges to adopting a comparative framework grounded in individual-based approaches. The first is how to obtain fair measures of cognitive performance across species that avoid potential biases that may arise for their differences in morphology, physiology, behaviour and ecology. One possibility is to make the comparisons within particular lineages or among distant species that share similar niches or coexist in similar environments. Webster & Lefebvre [75], for instance, investigated differences in neophobia, habituation and problem-solving among five avian species that forage together in Barbados. Experimental tests conducted both in the field and in captivity consistently showed that grackles and bullfinches outperformed pigeons and doves across all tasks—findings that align with their higher levels of encephalization and greater foraging opportunism [74–76].

A second challenge is that, while comparisons involving a small number of species can provide valuable insights, retrospective phylogenetic analyses require large sample sizes that capture sufficient trait variation, reduce the risk of overfitting and ensure robust and reliable inferences. Although characterizing cognitive performance across many species is challenging, several notable and ambitious efforts have been made. Examples include captive studies on neophobia and prosociality in corvids [77,78], exploration in parrots [70], problem-solving in carnivores [79] and birds [80], and inhibitory behaviour across birds and mammals [81].

As discussed in previous sections, assessing cognitive performance is only a first step in determining whether a cognitive trait is an adaptation; it is equally crucial to evaluate the trait’s heritability, its impact on individual fitness, and its broader demographic and evolutionary consequences. For example, if similar selection gradients are observed across multiple species in comparable environmental conditions, this would suggest convergent selection pressures, strengthening the case for the trait’s adaptive value. Conversely, variation in selection gradients across species or environments could suggest context-dependent selection, which also informs our understanding of adaptation and constraint.

While a comparative framework based on individual-level data could provide a more comprehensive understanding of cognitive evolution, it demands large-scale, coordinated efforts. Encouragingly, recent initiatives in comparative psychology—such as the *Big Team Science* projects (e.g. ManyPrimates [82], ManyBirds [83])—demonstrate that such collaborative approaches not only are feasible but also hold great potential for advancing the field. If a ‘ManyWilds’ collaborative team could be formed, this would facilitate the adoption of a shared conceptual framework, promote the use of consistent and more refined methodologies, and enable reciprocal validation of predictions across different levels of analysis (i.e. individual, population, species and lineage). Even if this framework were applied to only a small number of closely related species, it would still represent a transformative step towards uncovering the mechanisms underlying cognitive evolution.

## 9. Concluding remarks

Individual-based studies are essential for understanding the evolutionary origin and adaptive function of cognition. By tracking the development and life histories of identifiable animals, researchers can examine how cognitive abilities contribute to the survival and reproduction of individuals in their environments. Identifying the fitness benefits of cognitive traits helps determine whether a population is likely to persist and how well it is adapted to current conditions. When paired with information on the heritability of these traits, such studies allow us to assess their evolutionary potential and predict the conditions under which cognitive evolution may occur. Integrating data across populations and species improves the generalizability of the findings, and offers a retrospective lens through which to evaluate whether cognition actually evolved under diverse selective pressures.

The adoption of an individual-based approach has greatly advanced our understanding of the adaptive basis of cognition. As highlighted in this and other papers within this special issue, there is growing evidence that certain cognitive traits exhibit moderate heritable variation and confer fitness benefits, supporting their potential to evolve. However, progress remains incomplete without evidence of how cognition influences fitness, and how this in turn affects population growth and drives adaptive change—evidence that is currently limited. We believe this gap arises from a narrow focus on the challenges of identifying and quantifying cognitive performance, while overlooking the complexities of demographic and evolutionary processes. Such a narrow focus has sometimes led to the estimation of fitness components based on convenience rather than their relevance to overall fitness, an underappreciation of the need for large sample sizes, and less focus on cognitive traits as part of the entire phenotype. Additionally, much of the past research has relied on short-term data, limiting the ability to assess microevolutionary trends. Without a broader perspective, we risk overlooking the fitness consequences of cognition by focusing on fitness components that exhibit little variation or neglecting the critical gap of connecting microevolutionary processes with macroevolutionary patterns. Bridging this latter divide requires a more systematic integration of individual-based studies with comparative, cross-species analyses. Such an integrative framework would not only enhance scientific rigour and open new research directions, but also hold great promise for uncovering the drivers and constraints that have shaped the extraordinary diversity of minds across the animal kingdom. With continued efforts to improve and broaden the individual-based approach, the future of the field looks promising, offering exciting opportunities for a deeper and more unified understanding of the evolution of animal cognition.

## Data Availability

This article has no additional data.
